# Exploring differences in healthcare utilization of prisoners in the Canton of Vaud, Switzerland

**DOI:** 10.1371/journal.pone.0187255

**Published:** 2017-10-30

**Authors:** Karine Moschetti, Véra Zabrodina, Pierre Stadelmann, Tenzin Wangmo, Alberto Holly, Jean-Blaise Wasserfallen, Bernice S. Elger, Bruno Gravier

**Affiliations:** 1 Institute of Social and Preventive Medicine, University of Lausanne and University Hospital of Lausanne (CHUV), Lausanne, Switzerland; 2 Technology Assessment Unit, University Hospital of Lausanne (CHUV), Lausanne, Switzerland; 3 Institute for Biomedical Ethics, University of Basel, Basel, Switzerland; 4 Institute of Health Economics and Management, HEC Lausanne, University of Lausanne, Lausanne, Switzerland; 5 University Centre of Legal Medicine, University of Geneva, Geneva, Switzerland; 6 Service of Correctional Medicine and Psychiatry, University Hospital of Lausanne (CHUV), Lausanne, Switzerland; University of North Carolina at Chapel Hill, UNITED STATES

## Abstract

Prison healthcare is an important public health concern given the increasing healthcare needs of a growing and aging prison population, which accumulates vulnerability factors and suffers from higher disease prevalence than the general population. This study identifies the key factors associated with outpatient general practitioner (GP), nursing or psychiatric healthcare utilization (HCU) within prisons. Cross-sectional data systematically collected by the prison medical staff were obtained for a sample of 1664 adult prisoners of the Canton of Vaud, Switzerland, for the year 2011. They contain detailed information on demographics (predisposing factors), diagnosed chronic somatic and psychiatric disorders (needs factors), as well as prison stay characteristics (contextual factors). For GP, nurse and psychiatric care, two-part regressions are used to model separately the probability and the volume of HCU. Predisposing factors are generally not associated with the probability to use healthcare services after controlling for needs factors. However, female inmates use higher volumes of care, and the volume of GP consultations increases with age. Chronic somatic and psychiatric conditions are the most important predictors of the probability of HCU, but associations with volumes differ in their magnitude and significance across disease groups. Infectious, musculoskeletal, nervous and circulatory diseases actively mobilize GP and nursing staff. Schizophrenia, illicit drug and pharmaceuticals abuse are strongly positively associated with psychiatric and nurse HCU. The occupancy rate displays positive associations among contextual factors. Prison healthcare systems face increasingly complex organizational, budgetary and ethical challenges. This study provides relevant insights into the HCU patterns of a marginalized and understudied population.

## Introduction

Healthcare provision in prisons has recently been receiving much attention from public health stakeholders. Prison health services are responsible for ensuring sufficient access and levels of quality of healthcare services to meet prisoners’ health needs. These needs are substantial and rising, as the prison population is growing worldwide [1,2], with a greater increase in the relative share of elderly inmates [3,4]. While prison health systems are subject to ever tighter budgetary restrictions and pressed to optimize their efficiency, healthcare provision in prisons involves complex logistics. Understanding the patterns of healthcare utilization (HCU) in prisons is therefore of high public health and policy relevance.

The characteristics of inmates and their environment partly explain why HCU is found to be greater in prison than in the general population [5–8]. Inmates accumulate socioeconomic vulnerability factors such as chaotic life experiences, unemployment, low educational backgrounds, or lack of health insurance. Epidemiological evidence shows that prisoners suffer from higher disease prevalence than the general population [1,9–12], with psychiatric disorders, substance abuse and infectious diseases being particularly widespread [1,12–15]. Furthermore, health and HCU in prisons may be affected by context-specific factors, such as anxiety, sedentariness, isolation, self-harm, confined environments or hope to obtain psychotropic medications, but also organizational constraints, such as limited opportunities for health self-managment and lack of access to informal care [7,16–18].

Several studies have focused on explaining the variation in HCU across prisoners. Among personal characteristics, older age is found to be associated with HCU [5,19–22]. Some studies show higher HCU for female inmates [6,19], while others find no association [22,23]. The effects of race and ethnicity remain unclear [5,22,24]. Further research reveals self-reported health status and conditions, substance abuse and HCU prior to incarceration to be associated with HCU in prisons [19,21,25]. A recent meta-analysis focusing on adjustment to life in prison reports sufficient evidence for age, physical symptoms, prior mental health treatment and substance abuse being systematically associated with HCU [24]. These conclusions are nevertheless based on a small number of studies, which underlines the scarcity of the evidence on HCU in prisons. In light of this, additional research is necessary to understand the variation in HCU among prisoners.

This study identifies the epidemiological, demographic, and contextual factors associated with outpatient general practitioner (GP), nurse and psychiatric HCU in prisons. We use rich data systematically collected by the prison medical staff that incude information on individual prisoners’ HCU, chronic somatic and psychiatric conditions, as well as prison stay characteristics of the detainees of the Canton of Vaud in Switzerland. Our article makes several contributions to the literature above. First, we discriminate between specialties among the most consulted medical professionals in a prison setting. Second, our dataset contains specific conditions diagnosed and reported (more) objectively by physicians. This detailed account allows us to adjust for the complete chronic disease profile of the prisoner when modelling HCU. Meanwhile, existing studies have mainly relied on descriptive statistical analyses that do not sufficiently adjust for confounding epidemiological factors, or used generic measures of health status self-reported by the prisoner (e.g. self-assessed health or perceived quality of life). Third, our methodology uses two-part regression models to assess potentially differing determinants of the probability of using any healthcare on the one hand, and higher consultations frequency on the other hand. Hence, beyond the usual outcomes constructed as binary variables indicating if the inmate used any healthcare or not, we also capture HCU volumes.

Although we cannot make strong causal claims regarding the effects of the factors considered, this analysis provides valuable insights into the HCU patterns of a population that is marginalized and usually excluded from health-related population studies or surveys, and whose needs in terms of healthcare services are understudied. Our results can be combined with epidemiological evidence on disease prevalence to inform the organization of the three main areas of care provision in prisons.

## Methods

### Setting

In Switzerland, healthcare service provision in prisons is governed by the medical and ethical guidelines of the Swiss Academy of Medical Sciences [26], under the principle of equivalence of care established by international norms [27,28]. Prisoners must have access to the same quality of care as the general population, and the State is responsible for the health of imprisoned individuals. The Swiss prison health system is decentralized by canton (state), meaning that there are 26 distinct systems that may differ substantially in their legal and organisational frameworks.

In the Canton of Vaud, healthcare service provision inprison is independent of the judiciary authorities since 1995 [29]. Each prison has an on-site outpatient clinic managed by the Service of Correctional Medicine and Psychiatry (SMPP) of the University Hospital of Lausanne (CHUV). These clinics are the main points of provision of healthcare services to prisoners. Prison staff and healthcare professionals are civil servants and prisons are entirely public facilities. Standard healthcare services are provided at low or even zero financial cost for the prisoners. Basic health insurance is mandatory for all residents of Switzerland, including incarcerated individuals, and is acquired through a private health insurance company (third-party payer). Prisoners with sufficient means must hence continue purchasing their own insurance, which covers the expenditures for healthcare services in prison. The prison administration steps in to purchase insurance or cover healthcare expenditures for inmates who cannot conclude an insurance contract themselves (e.g. lack of financial resources or irregular legal status).

**Fig 1:**
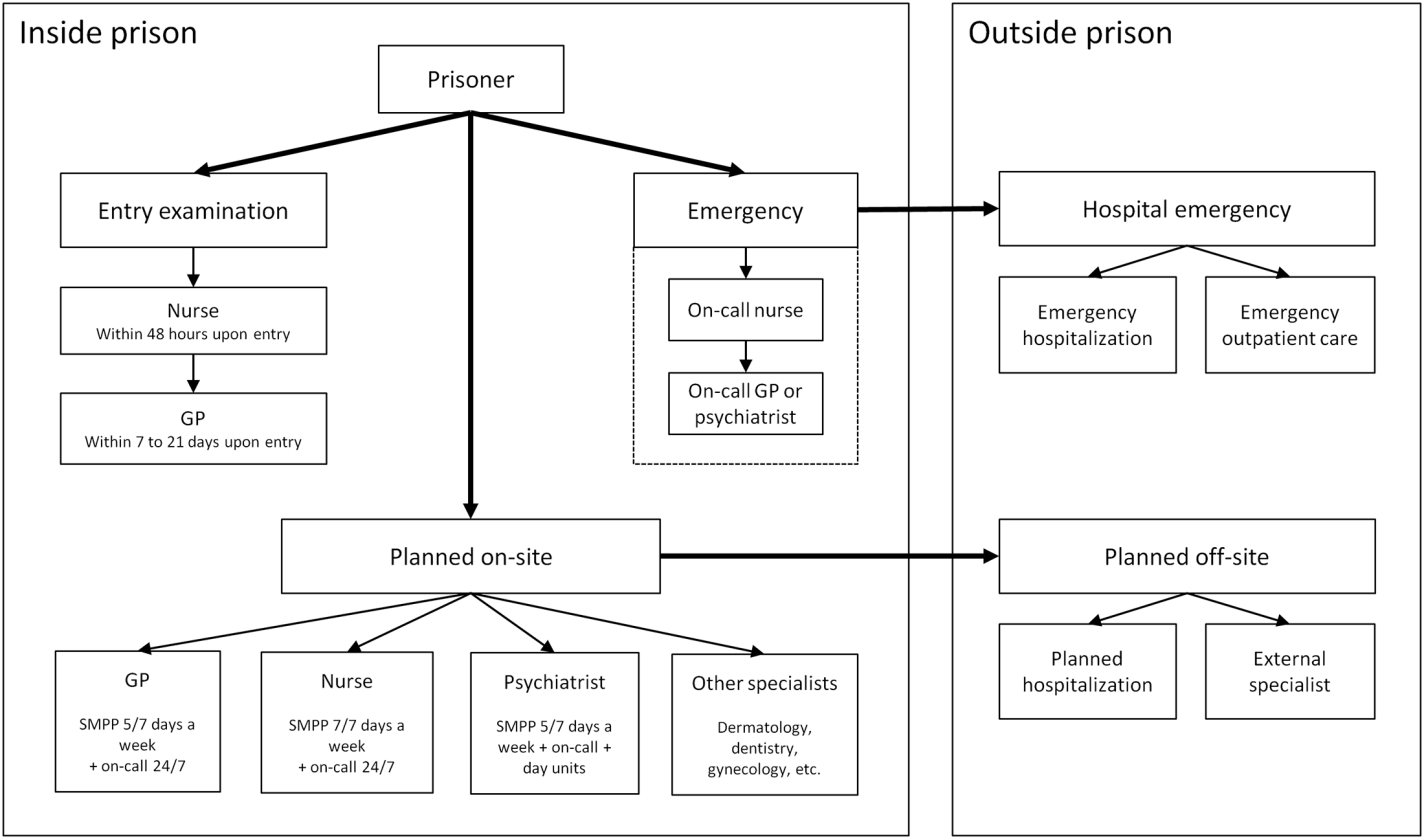
Prisoner management and access to healthcare services in the canton of Vaud. SMPP: Service of Correctional Medicine and Psychiatry (Service de Médecine et Psychiatrie Pénitentiaires).

Fig 1 summarizes inmates’ pathways to healthcare services in the Canton of Vaud. Upon prison entry, the prisoner is systematically seen by a nurse within 48 hours, and by a GP within 7 to 21 days. These entry examinations ensure a thorough screening for somatic—including infectious—diseases as well as a preliminary psychiatric evaluation. They may not be conducted if the prison stay is particularly short or if the prisoner refuses them. Beyond scheduled follow-up consultations, inmates may access healthcare by submitting a written application to the prison medical services, or through a request made by lawyers, prison officers, social agents or even other prisoners. Nurses and GPs are generally the first point of medical contact for prisoners, including emergencies outside working hours. They act as gatekeepers for access to external specialists and inpatient care, which involves transfers requiring authorizations and coordination with police and hospital security. Prison guards are responsible for accompanying detainees toall consultations or transfers.

### Data

This study uses cross-sectional data on the 1664 adult inmates who were incarcerated in the closed prisons of the canton of Vaud at any point in 2011 and who had a medical examination upon entry (see [10] for more details on the dataset). The data contain detailed information on HCU, demographics, chronic diseases and contextual factors systematically collected by the prison medical staff.

### Variables

Our modelling of HCU is based on Aday and Andersen’sframework of access to healthcare [30]. Our study focuses on the amount of realized access to healthcare and adapts the models of individual HCU to our specific setting. Three groups of predictors of HCU are retained: 1) Predisposing factors, which capture pre-existing individual propensities to use health services and generally include individual demographic characteristics. 2) Needs factors, which capture the health status as perceived by the individual or as evaluated by the provider. 3) Contextual factors, which capture the organization of the prison and its healthcare system, as well as individual detention conditions. Finally, in contrast to the standard model, the legal and institutional particularities of the correctional system neutralize the direct effect of enabling factors (e.g. socioeconomic variables related to the ability to access healthcare) on HCU. This statement applies to the prison healthcare system in the canton of Vaud, and may not necessarily be generalized to all Swiss cantons. Nevertheless, enabling factors may have affected the prisoner’s living conditions and access to healthcare prior to incarceration, and therefore health status upon incarceration.

#### Dependent variables: Measures of healthcare utilization

We focus on the three main types of healthcare services provided on-site to prisoners, namely GP, nurse, and psychiatric care. For each of three types of HCU, we construct two outcome variables: a binary variable indicating use versus non-use, and a count variable indicating the number of consultations for those inmates with at least one consultation, namely the volume of HCU. This distinction will allow us to model the decision to use healthcare and the intensity of use separately, and to identify potentially differing influences of independent variables. The total number of consultations is considered, even when the detainee had multiple stays over the year 2011. The entry examinations imposed by the legal framework are registered separately in the data and excluded from the total number of consultations since they are outside the strict individual demand for care, as well as a necessary step to establish the detainee’s health profile. Except for the entry examination, all consultations are included, whether the expenses are covered by health insurance or prison administration.

#### Independent variables

Predisposing factors include indicators for age groups, gender, origin (Swiss or African, with other as the reference) and being married. The needs factors are proxied byindicators for chronic somatic and mental health conditions, which are reported using the International Classification of Diseases, version 10 (ICD-10). As previously established in an epidemiological study of disease prevalence using this sample of prisoners [10], somatic conditions are sorted into the following groups: infectious (HIV, hepatitis B and C), skin, musculoskeletal, digestive, circulatory, endocrine, respiratory and nervous system. Mental health conditions are grouped as schizophrenia, mood disorders, neurotic disorders, behavioural syndrome, personality disorder and mental retardation. Substance abuse problems include alcohol, illicit drug and pharmaceuticals abuse. Furthermore, to capture the indirect influence of enabling factors, we include an indicator for having health insurance upon incarceration as an additional measure of health, rather than a factor directly enabling HCU within the prison.

Additionally, both personal and prison-specific detention conditions may impact the way individuals behave, adjust to life in prison, and use healthcare. Consequently, we adjust for the following contextual factors: type of crime (violent, sexual or drug-related—either selling or consumption—with other crimes as the reference), detention regime (preventive or convicted), number of stays in 2011, as well as the mean occupancy rate of the prison during the prisoner’s stay. In case of multiple stays, the mean occupancy rate is weighted by length of stay and averaged across stays. We also adjust for the length of stay, as the time dimension is directly related to the opportunity to use healthcare: the longer the prison stay, the greater the opportunity to use healthcare services. Finally, prison indicators are included to account for potential heterogeneities in the structural characteristics of the care units in each prison but also of the prison facility itself more broadly that may influence access to healthcare and HCU.

### Statistical analyses

Descriptive statistics are presented for HCU, predisposing, needs and contextual factors to examine the characteristics of the sample (Table 1). Then, GP, nurse and psychiatric HCU are modelled separately using two-part models. These models are commonly used to analyze HCU as they account for the mass of observations with zero utilization by distinguishing individuals with no HCU from those with strictly positive volumes of HCU [31]. First, we model the probability to use healthcare using logistic regressions, with a logarithmic link and a binomial distribution. Second, the number of consultations is modelled for detainees with any HCU using zero-truncated negative binomial regressions with a logarithmic link. The negative binomial distribution accounts for the count nature (non-negative integers), as well as the skewness and heavy-tailed distribution of the outcome variables [32]. The estimation is carried out with pseudo-maximum likelihood, which yields consistent estimates as long as the conditional mean of the dependent variable is correctly specified [33,34]. Two-part models allow for differences in the impact of specific individual characteristics on the probability to use versus the intensity of use of healthcare services. The probability to use healthcare services is assumed to be independent of the intensity of use, so that error terms between the two stages are uncorrelated. The total length of stay in 2011 is included as an exposure variable in both estimation stages. The statistical analyses were performed using Stata 14.

**Table 1:** Summary statistics of independent variables.

	% (N)
**Predisposing factors**	
Male^a^	91.5 (1524)
Female	8.5 (140)
Aged 18–29 ^a^	47.0 (782)
Aged 30–39	29.9 (497)
Aged 40–49	15.0 (249)
Aged 50 and older	8.2 (136)
Not married^a^	80.8 (1348)
Married	19.2 (316)
Other origin^a^	55.6 (925)
Swiss origin	20.0 (332)
African origin	24.4 (406)
No health insurance^a^	55.0 (916)
Has health insurance	45.0 (748)
**Needs factors**	
*Chronic somatic conditions*	
Infectious diseases	8.9 (149)
Musculoskeletal system	12.8 (213)
Nervous system	1.4 (23)
Circulatory system	6.5 (108)
Skin problems	8.1 (135)
Digestive system	7.6 (127)
Respiratory system	5.5 (91)
Endocrine system	5.1 (85)
*Mental health conditions*	
Schizophrenia	5.3 (88)
Mood disorders	2.2 (37)
Neurotic disorders	15.9 (264)
Behavioral syndromes	1.9 (31)
Personality disorders	16.2 (269)
Mental retardation	2.8 (46)
*Substance abuse problems*	
Alcohol	9.5 (159)
Illicit drugs	18.0 (298)
Pharmaceuticals	5.2 (87)
**Contextual factors**	
Other type of crime^a^	57.5 (955)
Violent crime	5.0 (83)
Sexual crime	5.8 (97)
Drug-related crime	31.7 (529)
Convicted^a^	61.1 (1017)
Preventive detention	38.9 (647)
Number of stays in 2011^b^	1.23 (0.5)
Length of stay in 2011 (in days)^b^	132.5 (121.5)
Total length of stay (in days)^b^	290.5 (559.3)
Mean prison occupancy rate (in %)^b^	113.7 (24.0)
Stayed in Bois-Mermet prison	33.5 (558)
Stayed in Plaine d'Orbe prison	24.4 (406)
Stayed in Croisée prison	43.3 (721)
Stayed in La Tuilière prison	13.3 (221)
*N*	1664

^a^ Reference categories in regression models.

^b^For continuous variables, the table reports the mean (SD).

### Ethical considerations

The study was approved by the ethical commission of the Canton of Vaud, Switzerland (Protocol No. 388/12). As the study was retrospective, used anonymized data and displayed aggregated results only, individual agreement was not required.

## Results

### Descriptive statistics

Table 1 describes the independent variables, namely predisposing, needs and contextual factors. The sample includes only 8.5% of female prisoners, and almost half of the sample is younger than 29 years old. Only 20% of detainees are of Swiss origin, about 24% are from Africa, with other inmates primarily from Eastern Europe. The demographic profile of our sample is similar to the nationwide statistics on the Swiss prison population [35]. Only 45% of detainees have health insurance.

In our sample, 40.6% of detainees have at least one diagnosed chronic somatic condition, 44.8% have at least one psychiatric condition. The most prevalent chronic disease groups are infectious (8.9%) and musculoskeletal (12.8%) among somatic conditions, neurotic (15.9%) and personality disorders (16.2%) among psychiatric conditions, and illicit drugs (18.0%) among substance abuse problems. Only 37.0% of detainees have neither a chronic somatic nor a psychiatric condition. The average length of stay over 2011 was 132.5 days (SD = 121.5), with a median at 85 days, while the average total length of stay was of 290.5 days and displayed higher variation (SD = 559.3).

Table 2 shows descriptive statistics for the three measures of HCUfor the whole sample of detainees, and the subsample of those with at least one diagnosed chronic condition. Looking at the whole sample, detainees had 2.5 GP consultations on average (including those with no consultations), with 0.6 consultations per month on average. Almost 82% had at least one GP consultation. Nursing visits were most frequent, at an average of 11.6 absolute visits, 2.6 per month and 90% of detainees with at least one visit. As for psychiatrists, the absolute and monthly average number of consultations was similar to GPs, but only 43% of detainees had at least one consultation. Detainees with at least one diagnosed chronic condition display higher averagesand dispersion for all three types of care of HCU, and higher proportions of inmates with at least one follow-up consultation. The data also show that visits to GPs, nurses and psychiatrists amount to 89% of all consultations made on-site (including other medical specialties).

**Table 2:** Summary statistics of generalist, nursing and psychiatric healthcare utilization.

	Mean	25th percentile	Median	75th percentile	Inmates with at least one consultation (%)	Mean consultations per 30 days of incarceration
Whole sample of inmates (*N* = 1664)						
GP consultations	2.5	1	1	3	81.9	0.6
Nurse consultations	11.6	2	7	15	89.9	2.6
Psychiatrist consultations	2.7	0	0	3	43.2	0.6
Inmates with at least one chronic somatic or psychiatric health condition (*N* = 1048)						
GP consultations	3.3	1	2	4	91.6	0.6
Nurse consultations	16.8	5	11	21	97.7	2.9
Psychiatrist consultations	4.6	0	2	5	66.1	0.8

All statistics exclude the examination upon prison entry (see Methods section).

### Results of two-part regression models

Table 3 presents the results of the estimated two-part regression models for generalist, nursing and psychiatric HCU.

**Table 3:** Results of two-part regression models of prisoners’ generalist, nursing and psychiatric healthcare utilization.

	General practitioners				Nurses				Psychiatrists			
	Logistic regression		Zero-truncated negative binomial regression		Logistic regression		Zero-truncated negative binomial regression		Logistic regression		Zero-truncated negative binomial regression	
	OR	SE	IRR	SE	OR	SE	IRR	SE	OR	SE	IRR	SE
**Predisposing characteristics**												
Female	1.47	(0.82)	1.38***	(0.17)	3.34	(4.14)	1.36**	(0.17)	0.26*	(0.21)	1.36*	(0.22)
Aged 30–39	0.88	(0.18)	1.02	(0.05)	0.77	(0.19)	1.02	(0.06)	1.14	(0.26)	0.92	(0.07)
Aged 40–49	0.96	(0.25)	1.14*	(0.08)	1.27	(0.49)	0.98	(0.07)	1.31	(0.36)	0.90	(0.09)
Aged 50 and older	0.61	(0.25)	1.21**	(0.12)	0.62	(0.33)	1.11	(0.12)	0.72	(0.30)	0.72**	(0.10)
Married	1.04	(0.24)	0.97	(0.05)	1.43	(0.46)	1.00	(0.06)	0.52**	(0.14)	0.95	(0.09)
Swiss origin	1.21	(0.32)	1.10	(0.07)	1.40	(0.49)	1.19***	(0.08)	1.60*	(0.43)	1.28***	(0.10)
African origin	1.20	(0.26)	0.90*	(0.05)	1.67*	(0.48)	0.97	(0.07)	0.33***	(0.08)	0.71***	(0.08)
Has health insurance	1.16	(0.22)	1.00	(0.05)	1.22	(0.30)	1.10*	(0.06)	1.67***	(0.32)	1.02	(0.07)
**Needs factors**												
*Chronic somatic conditions*												
Infectious diseases	12.17***	(8.13)	1.17**	(0.08)	3.85	(3.18)	1.19**	(0.09)	2.16***	(0.59)	0.98	(0.09)
Musculoskeletal system	27.40***	(21.14)	1.13**	(0.06)	9.79***	(8.55)	1.05	(0.06)	1.22	(0.30)	0.90	(0.07)
Nervous system	-	(.)	1.40***	(0.18)	-	(.)	1.49***	(0.23)	4.29**	(3.17)	1.42	(0.37)
Circulatory system	14.53***	(11.45)	1.25***	(0.11)	9.81***	(8.17)	1.62***	(0.14)	0.79	(0.31)	1.01	(0.13)
Skin problems	2.96**	(1.46)	1.09	(0.08)	1.92	(1.33)	0.96	(0.07)	0.54	(0.22)	1.00	(0.11)
Digestive system	6.14***	(3.75)	0.95	(0.07)	3.79*	(3.07)	0.93	(0.07)	0.84	(0.30)	1.18*	(0.12)
Respiratory system	3.36**	(1.99)	1.07	(0.09)	5.34	(5.98)	1.01	(0.09)	1.29	(0.51)	0.70***	(0.08)
Endocrine system	4.19	(3.71)	1.13	(0.10)	2.68*	(1.49)	1.08	(0.10)	1.26	(0.52)	1.10	(0.14)
*Mental health conditions*												
Schizophrenia	0.68	(0.29)	0.88	(0.12)	2.02	(1.74)	1.40***	(0.18)	47.28***	(34.51)	2.37***	(0.27)
Mood disorder	0.52	(0.32)	1.06	(0.15)	0.81	(0.95)	1.09	(0.12)	27.66***	(20.47)	1.21	(0.18)
Neurotic disorder	2.99***	(1.03)	0.99	(0.06)	2.92*	(1.66)	1.11*	(0.06)	88.22***	(35.59)	1.03	(0.08)
Behavioral syndrome	0.80	(0.49)	0.87	(0.12)	1.35	(1.36)	0.79**	(0.09)	18.10***	(11.47)	0.73*	(0.13)
Personality disorder	0.68	(0.21)	0.92	(0.06)	1.03	(0.57)	1.00	(0.06)	7.29***	(2.66)	1.15*	(0.09)
Mental retardation	1.51	(1.03)	0.98	(0.11)	1.76	(2.19)	1.24**	(0.12)	52.73***	(51.25)	1.15	(0.12)
*Substance abuse problems*												
Alcohol	1.27	(0.44)	1.06	(0.08)	1.15	(0.65)	1.07	(0.07)	17.07***	(8.64)	1.08	(0.08)
Illicit drugs	1.58	(0.50)	0.97	(0.06)	4.33**	(2.68)	1.27***	(0.08)	22.67***	(8.75)	1.28***	(0.10)
Pharmaceuticals	1.39	(0.68)	0.97	(0.08)	2.83	(3.35)	1.35***	(0.12)	9.37***	(7.76)	1.23**	(0.11)
**Contextual factors**												
Violent crime	1.16	(0.69)	0.81*	(0.09)	2.36	(2.45)	0.84	(0.10)	1.14	(0.53)	0.85	(0.11)
Sexual crime	1.62	(0.70)	0.80**	(0.08)	1.43	(1.06)	0.94	(0.09)	1.48	(0.64)	1.08	(0.12)
Drug-related crime	0.56***	(0.12)	0.86***	(0.05)	0.44***	(0.12)	0.75***	(0.04)	0.90	(0.21)	0.87*	(0.07)
Preventive detention	1.54*	(0.39)	0.82***	(0.05)	2.05**	(0.67)	0.87**	(0.06)	0.71	(0.17)	1.13	(0.09)
Number of stays in 2011	1.09	(0.30)	0.99	(0.06)	1.88	(0.92)	1.05	(0.06)	1.19	(0.34)	1.00	(0.08)
Total length of stay	1.00***	(0.00)	1.00***	(0.00)	1.00***	(0.00)	1.00**	(0.00)	1.00***	(0.00)	1.00***	(0.00)
Mean prison occupancy rate	1.01	(0.01)	1.01***	(0.00)	1.01	(0.01)	1.01***	(0.00)	1.01	(0.01)	1.01***	(0.00)
Stayed in Bois-Mermet prison	0.62	(0.30)	0.92	(0.09)	0.89	(0.79)	0.73***	(0.08)	0.40	(0.23)	0.77*	(0.11)
Stayed in Plaine d'Orbe prison	0.46*	(0.21)	0.82*	(0.09)	2.79	(2.72)	0.86	(0.09)	0.40*	(0.20)	1.15	(0.18)
Stayed in Croisée prison	2.21*	(0.96)	0.81**	(0.08)	3.75	(3.37)	1.07	(0.10)	0.52	(0.24)	1.47***	(0.20)
Stayed in La Tuilière prison	1.08	(0.57)	0.96	(0.10)	8.48*	(10.50)	1.05	(0.11)	1.34	(0.71)	1.35*	(0.22)
Constant	0.02***	(0.02)	0.01***	(0.00)	0.01***	(0.01)	0.04***	(0.01)	0.00***	(0.00)	0.01***	(0.00)
Observations	1664		1363		1664		1495		1664		718	

OR: odds ratio (exponentiated coefficient of the regression); IRR: incidence rate ratio (exponentiated coefficient of the regression); SE: standard error. Robust standard errors in parentheses. Aged 18–29, male, convicted, other origin and other type of crime are reference categories. All regressions include the total length of stay over 2011 as an exposure variable. All detainees with nervous system disorders consulted generalist practitioners and nurses, so that the corresponding indicator predicts success perfectly.

* *p* < 0.1,

** *p* < 0.05,

*** *p* < 0.01.

#### Predisposing factors

Gender and age are generally not associated with the probability of HCU, except for female inmates having a lower probability of using psychiatric care. However, female inmates display significantly higher volumes for all three types of HCU, all else being equal. Age is not associated with the probability of using healthcare, but the volume of GP visits increases with age and individuals older than 50 use significantly less psychiatric care. Being married has no influence on GP or nursing consultations, but is associated with a lower probability of using psychiatric care. Detainees of Swiss origin use larger volumes of nurse and psychiatric care than detainees from other origins. Detainees of African origin have significantly lower psychiatric HCU. Finally, having health insurance is only significantly associated with higher volumes of nursing care, and higher probabilities to use psychiatric care.

#### Needs factors

Chronic somatic diseases are—with few exceptions—positively associated with both the probabilities and volumes of GP and nursing HCU. However, the associations differ in their magnitude and significance across disease groups. Infectious diseases significantly increase both the probability and the volume of GP HCU. They are also associated with a higher volume of nursing care, and likelihood of using psychiatric care. Diseases of the musculoskeletal system increase GP and nurse HCU, as do nervous and circulatory system diseases. While skin, digestive and respiratory diseases increase the probability of using generalist care, they do not display significant relationships with the volume of consultations. No associations emerge for endocrine conditions, except for a higher probability of using nursing care.

Generally, psychiatric conditions and substance abuse disorders have significant positive associations with both the probability and the volume of psychiatric care, but do not influence GP HCU. However, schizophrenia, neurotic disorders, mental retardation, as well as illicit drug and pharmaceuticals abuse have positive relationships with the volume of nurse visits.

#### Contextual factors

No systematic pattern emerges for types of crime across the models of HCU, except for drug-related crimes being associated with lower probabilities and volumes of GP and nurse HCU than other types of crime. Being in preventive detention is associated with higher probabilities, but lower volumes of GP and nurse HCU. The mean occupancy rate is highly significantly associated with the volume of HCU for all three outcomes, although the magnitude of the association is small. Previous stays in prison do not influence HCU. Total length of stay is consistently significantly negative, after adjusting for the length of stay in 2011 as an exposure variable, but the difference from 1 is small (OR and IRR equal to 0.998–0.999, rounding up to 1.00 in Table 3). Finally, prison indicators are significant in some models. While staying in the Bois Mermet prison is associated with lower volume of nursing and psychiatric HCU, being incarcerated in La Croisée or in La Tuilière is associated with agreater probability of consulting a GP or a nurse as well asvolume of psychiatric visits.

## Discussion

With regards to predisposing factors, our finding of no significant differences in the probability of using healthcare between male and female prisoners—after adjusting for chronic conditions—is consistent with previous studies analysing the probability of HCU only [22,23]. However, our results show significantly higher volumes of HCU for female inmates, similarly to Lindquist and Lindquist [19]. This result is aligned with the epidemiological evidence underlining the larger disease burden and specific needs of female prisoners [10,36].

Chronic somatic and psychiatric conditions are highly correlated with age, so that the disease indicators partly capture the effect of age-related morbidity. Still, since chronic conditions do not reflect the full health profile of the individual, age may partly capture morbidity or frailty. This may explain the higher volumes of GP consultations among older prisoners, as found in the literature. However, the consumption of psychiatric care is lower among older inmates, despite higher prevalence of mental health disorders in this group [10]. A possible explanation is that age captures unobservable factors not fully captured by our models, such as having adjusted to life in prison through longer sentences, or having greater stability in the management of their mental health conditions. These factors may lead older detainees to seek or require less psychiatric care. Previous studies have nonetheless underlined the need to better identify mental health issues specific to older prisoners [37]. Finally, our results show disparities across origins, with higher volumes of nurse and psychiatric HCU for Swiss inmates. This may be due to a better understanding of the prison healthcare system or more easily identified needs through the absence of language or cultural barriers.

In the studied prisons unlike in the general population, access to healthcare services does not depend on the individual’s financial resources. This feature refers to a general policy objective in many countries with publicly financed healthcare systems: horizontal equity in healthcare delivery, which implies equal treatments for equal needs irrespective of non-needs characteristics and ability to pay [38]. Indeed, our results tend to show equity among prisoners, with predisposing factors and insurance generally not associated with HCU after controlling for needs factors. These findings also suggest that part of the variation in HCU usually attributed to predisposing factors is in fact explained by chronic conditions, and underline the importance of accounting for the confounding effect of chronic health conditions in studying HCU patterns.

Needs factors proxied by chronic somatic and psychiatric diseases are generally strong predictors of the probability to use healthcare, so that detainees with chronic diseases can be expected to have strictly positive HCU during incarceration. In terms of consultation volumes however, our results unveil that associations differ in terms of magnitude and significance. These relative differences in consultation frequencies across disease groupsmay be partly due to the needs for monitoring and the opportunities for self-management by prisoners. Among somatic conditions, infectious, musculoskeletal, nervous and circulatory diseases are associated with highervolumes of GP and nurse HCU. For example, hypertension requires frequent blood pressure checks and medication treatment. Infectious diseases may be asymptomatic but require careful somatic monitoring. Their significant impact on the probability of consulting a psychiatrist may be due to the high correlation between psychiatric conditions and infectious diseases, as inmates injecting drugs or suffering from psychiatric disorders are more prone to contamination prior or during incarceration [3,39]. Meanwhile, many skin, digestive, respiratory or endocrine diseases require less involvement from the medical staff. For instance, diabetic prisoners are sometimes allowed to monitor blood glucose levels themselves, explaining why the association between endocrine diseases and volumes of GP and nurse HCU is not significant.

Similar mechanisms are at play for psychiatric disorders and substance abuse problems. While some disorders, such as schizophrenia, or the abuse of illicit drugs or pharmaceuticals require regular contacts with nurses and psychiatrists to administer psychotropic medication or methadone substitution treatments, others, such as mental retardation, require less frequent monitoring. Neurotic disorders mainly encompass acute stress reactions, which may be associated with the shock of incarceration and explain their significant impact on the probability of all three types of HCU, and the volume of nursing HCU.

Aggregate effects on total HCU in the prison population depend both on the prevalence of the disease and its relative association with HCU. A general pattern in our results is that relatively prevalent chronic conditions—infectious and musculoskeletal diseases among somatic conditions; neurotic and personality disorders among psychiatric conditions; and illicit drug among substance abuse disorders—also emerge as significant predictors of HCU with relatively large IRRs. Other disease groups, despite lower prevalence, display high-magnitude ORs and IRRs in two-part models, e.g. nervous system diseases, schizophrenia or pharmaceuticals abuse.

In the community, many conditions can be managed through access to informal care such as pharmacy services or help of relatives. In prison however, they require regular contacts with qualified medical staff. A trade-off exists between safety and the autonomy given to the prisoner to self-manage conditions [40]. Potential for drug traffic or self-harm may limit the opportunities for autonomy, especially for detainees with psychiatric disorders. In this context, nurses have a fundamental role in providing daily care, delivering medication, but also in supporting prisoners. As the most consulted medical professionals, they are in a prime position to identify needs and redirect prisoners to relevant specialists. It is therefore important to plan enough nurse resources and to ensure context-specific training. More generally, opportunities to facilitate the management of chronic conditions and follow-up care in prisons should be further explored.

As for contextual factors, our results show no clear effect of total incarceration length and number of stays, after including the length of stay in 2011 as an exposure variable. Adjustment to life in prison is an important dimension for HCU, and counteracting influences may explain this result. In the short term, the shock of incarceration may be difficult to bear, especially for women [36]. It can for instance induce anxiety and sleeplessness [41], which can lessen with time. Prisoners in preventive detention are particularly exposed to problems related to sedentariness as they spend more than 20 hours a day in their cell, while convicted prisoners may have the opportunity to work and be more active. In addition, prisons may be the first point of access to healthcare for socioeconomically vulnerable detainees, leading to higher HCU upon incarceration [5]. In a longer term however, the length of incarceration may put more weight on the impact of contextual factors on somatic and mental health and generate additional needs, especially among older prisoners [20].

Another interesting finding is that the prison occupancy rate significantly increases HCU volumes. Overcrowding may exacerbate the psychological burden of incarceration, as well as facilitate the transmission of communicable diseases. It may also increase pressure on the medical staff and complicate effective liaisons with prison administration and officers. Finally, prison dummies beingsometimes associated with HCU points to unobserved prison-levelinfluences. These may encompass differences in health care unit organization across prisons, but also prison size, the composition of the prison population (e.g., preventive or long-term detention) and the availability of opportunities to work, meeting spaces and social activities for prisonners. These various factors may affect access to but also the prisoners’ needs for medical care. For instance, a pyschiatric day unit, as available in La Tuilière prison, may facilitate the use of psychiatric care. Furthermore, opportunitiesfor social interactions (e.g., sports activities or or access to spaceswhere inmates can socialize) that exist in some prisons (e.g., Bois Mermet) may benefit prisoner health by alleviating certains symptoms associated with detention such as isolation, stress and anxiety and thus may reduce the propension to visit healthcare providers. Conversely, facilities that do not favour social interactions, for instance due to their architectural construct, may exacerbate adjustment disorders and increase the need for care.

### Study limitations

This study has several limitations. First, despite a large set of physician-reported chronic disease indicators, we do not have a complete picture of health needs. Inmates also suffer from a wide range of acute problems such as injuries or self-harm that partly determine HCU. Second, concerns may arise about the endogeneity between HCU and the health profile of the inmate. Greater HCU may increase the probability of diagnosing chronic conditions that developed during incarceration, or prevent such conditions from arising. Focusing on chronic illnesses nevertheless limits these concerns in that the vast majority are already present at admission and average lengths of stay are short. Third, our analyses do not inform on potential unmet needs [12], or over-utilisation of healthcare services that systematically occur for given conditions, and rely on the assumption that the observed relationship between prisoner characteristics and HCU is correct. Similarly, the data do not allow us to identify whether the visit was planned or emergent,nor the type or quality of the specific healthcare services provided. Fourth, the data come from one canton and might not be fully representative of Switzerland. Other studies have shown noticeable differences in prison healthcare service provision both across Swiss cantons and internationally [42]. Finally, substitution or complementarities between the types of care are relevant dimensions for further research.

## Conclusion

This study identifies the key predisposing, needs and contextual factors associated with the utilization of outpatient GP, nursing and psychiatric care in prisons. Prison healthcare is an important public health concern, as the vast majority of inmates eventually re-enter the community. Adequate healthcare management during incarceration may improve health outcomes and rehabilitation upon release. Prison healthcare systems face increasingly complex organizational, budgetary and ethical challenges to cope with a growing and aging prison population. With this in mind, this study contributes to a deeper understanding of prisoners’ HCU patterns by identifying the individual characteristics that determine the probability and frequency of outpatient consultations with three main categories of medical professionals in prisons. In particular, this analysis identifiesseveral chronic somaticand psychiatric diseases associated with significantly higher relative probabilities and volumes of individual HCU, holding all other factors constant. Thereby, it provides potential targets for interventions aiming to improve disease management in prisons, facilitate access to follow-up care, or strengthen prevention (and thereby reduce pressure on medical staff). Furthermore, they can be combined with epidemiological evidence on disease prevalence in the prison population to inform the organisation of healthcare provision in prisons more broadly. Given the lack of access to informal care and autonomy, our results underline that nurses are an essential resource in prison health systems.
